# Computer-Aided Supporting Models of Customized Crack Propagation Sensors for Analysis and Prototyping †

**DOI:** 10.3390/s25020566

**Published:** 2025-01-19

**Authors:** Paulina Kurnyta-Mazurek, Rafał Wrąbel, Artur Kurnyta

**Affiliations:** 1 Faculty of Mechatronics, Armament and Aerospace, Military University of Technology, 00-908 Warsaw, Poland; 2 Airworthiness Division, Air Force Institute of Technology, 01-494 Warsaw, Poland; artur.kurnyta@itwl.pl; 3 Łukasiewicz Research Network-Institute of Aviation, 02-256 Warsaw, Poland; rafalwrabel@wp.pl

**Keywords:** parametric model, customized crack propagation sensor, COMSOL Multiphysics software, Abaqus software, rapid algorithm prototyping

## Abstract

The range of sensor technologies for structural health monitoring (SHM) systems is expanding as the need for ongoing structural monitoring increases. In such a case, damage to the monitored structure elements is detected using an integrated network of sensors operating in real-time or periodically in frequent time stamps. This paper briefly introduces a new type of sensor, called a Customized Crack Propagation Sensor (CCPS), which is an alternative for crack gauges, but with enhanced functional features and customizability. Due to those characteristics, it is necessary to develop a family of computer-aided supporting models for rapid prototyping and analysis of the new designs of sensors of various shapes and configurations, which this paper presents by use of simulation tools. For a prototyping of the sensor lay out, an algorithm is elaborated, based on an application created in LabVIEW 2022 software, which generates two spreadsheets formatted by the requirements of Autodesk Inventor 2014 and COMSOL Multiphysics 5.6 software, based on data entered by the user. As a result, a tailored-in-shape CCPS layout is prepared. A parametric model of the sensor is prepared in Autodesk Inventor software, which automatically changes its geometric dimensions after changing data in an MS Excel spreadsheet. Then, the generated layout is analyzed to obtain electromechanical characteristics for defined CCPS geometry and materials used in the COMSOL Multiphysics software. Another application is devoted to purely mechanical analysis. The graphical user interface (GUI) add-on based on the Abaqus 2018 software engine is prepared for advanced mechanical analysis simulations of sensor materials in selected loading scenarios. The GUI is used for entering material libraries and the selection of loading conditions and a type of specimen, while the results of the numerical analysis are delivered through Abaqus. The main advantage of the developed GUI is the capacity for personnel inexperienced in using the Abaqus environment to perform analysis. Some results of simulation tests carried out in both COMSOL Multiphysics as well as Abaqus software are delivered in this paper, using a predefined parametric sensor model. For example, using a rigid epoxy resin for an insulating layer shows a negligible difference in the level of strain compared to the structure during a simulated tensile test, specifically in the tested layer thickness range of up to 0.3 mm. However, during bending tests, an approx. 17% change in principal strain level can be observed through the top to bottom edge of the epoxy resin layer. The adopted methodology for carrying out simulation studies assumes the parallel use of a set of various computer-aided tools. This approach allows for taking advantage of individual software environments, which allows for expanding the scope of analyses and using the developed models and applications in further research activities.

## 1. Introduction

In the technical condition monitoring method, which is based on the concept of SHM (structural health monitoring) systems, the damage to a structural component is detected using an integrated sensor network operating in real time (e.g., during an aircraft flight) or used as a part of post-flight on-ground inspection. Airworthiness-related concepts could have a significant impact on the safety of the operational use of technical facilities between scheduled, periodic inspections, especially when access to many “hot spot” elements of a structure is difficult or even impossible. The utilization of such systems allows the detection of, e.g., fatigue cracks in the early phase of their development, before the damage reaches a critical dimension.

Several sensor technology candidates suitable for SHM application have been widely investigated at various stages of technology readiness level (TRL). Among these are guided wave excited piezoelectric transducers, e.g., lead zirconate titanate (also called PZT) ceramics [1,2], comparative vacuum monitoring sensors (CVM) [3], and foil eddy current sensors [4]. The work carried out in this article concerns the development of a new crack detection sensor, which is characterized by better operational properties than commercially available crack gauges solutions [5,6]. In order to reduce the scope of experimental studies, computer-aided methods were used to carry out additional research. The new type of crack gauge is named Customized Crack Propagation Sensor (CCPS) [7,8]. It could become a good alternative for crack detection and monitoring techniques, due to its economic and functional features discussed throughout this paper. Both the utilization of crack gauges and searches for new varieties of such a sensor can be found in the state-of-the-art literature. This includes the use of various insulating and electrically conductive materials [9,10], sensing grid shapes [11,12,13], and principles of operation [14,15]. All are dedicated to detecting surface fatigue cracks, which propagate under the sensor bonded to the host structure.

The CCPS is designed as a grid whose shape can be tailored using additive and extrusion-based manufacturing methods. However, the sensor shape customization and material engineering research that go into developing this new solution will benefit in using software-based approach and simulations. The algorithm developed within that work allows for automation of the CCPS sensor shape design process, parameterizing its characteristic geometric dimensions in individual layers. An additional advantage is the digital solid-type model of the sensor, which can be applied in other software environments using broadly understood finite element methods (FEM) analysis. During the simulation studies, two computational software packages were used: ABAQUS 2018 and COMSOL Multiphysics. These were selected due to their specific abilities and features in conducting numerical analysis.

Supporting experimental research with numerical analysis tools is a common method for expanding the scope of research, especially in the area of mechanical properties, including fatigue, electrical, thermal, and other properties. For example, in [16], FEM is used to study the behavior of the large deformation field near the tip of a crack in a soft incompressible plane stress fracture specimen loaded in the model. The ABAQUS software was used to solve the task for the strip specimen.

Another benefit of utilizing computer-aided methods is the ability to solve non-trivial problems. Within the scope of the following overall research, the CCPS sensor is adhesively bonded to the metallic structure on its surface. A similar task was conducted theoretically in [17]. A finite element method (FEM) model was built to validate the theoretical model. ANSYS software was used and plain-strain elements were adopted to solve the problem of formation of periodic crack patterns in a film-substrate bilayer structure. Meanwhile, in [18], a numerical approach was also employed to understand and compare the mechanical behavior of carbon-based epoxy adhesives. The cohesive zone model (CZM) was implemented in the ABAQUS finite element package, and that model was also used within the following research. The CZM, specified in terms of the traction-separation law, is a well validated method within FEM analysis. Its use was reported in [19], with its calibration procedure delivered in [20].

The content of this paper is organized as follows: in the first section, structural health monitoring systems are introduced. Next, the Customized Crack Propagation Sensor (CCPS) description is presented. The third section shows the rapid prototyping algorithm. Finally, theoretical studies with the use of COMSOL Multiphysics software are presented, and concluding remarks based on the achieved results are provided.

## 2. Customized Crack Propagation Sensors

The CCPS was developed as a new type of crack gauge, for which the measuring grid of the sensor should tear together with the monitored structure, due to the presence of a fatigue crack. This produces a change in CCPS output resistance, and that parameter can be acquired as a damage indicator for further analysis. The CCPS discussed in this paper can overcome the limitations and drawbacks of typical, commercially available crack gauges [21]. Commercial sensors of that type are usually unidirectional in crack propagation sensitivity, and the shape of the sensing grid is fixed, with only a few patterns available on the market.

**Figure 1:**
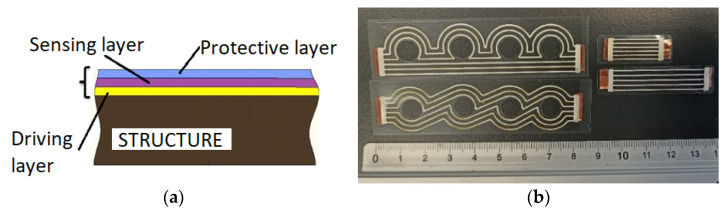
The Customized Crack Propagation Sensor: (**a**) cross-section; (**b**) real layout top view [6].

Meanwhile, the presented CCPS can be customized to the monitored component and allows tailoring of the shape of sensor measuring grid to the specific structure, even in the presence of dense riveting, holes, and surface curvature. This important feature of CCPS is possible due to the materials used in the functional layers to build the sensor and the way it is manufactured. CCPS are fabricated using flexible insulating materials for the driving and protective layers and conductive paste/ink with silver microparticles for the sensing layer (Figure 1). The manufacturing process includes thin film casting with a doctor blade for the driving layer, and selective direct writing with extrusion syringe-based deposition in the sensing and protective layers.

The feature of adapting the measuring grid shape translates directly into the ability to detect even relatively short cracks (sensor closer to the “hot spot”) and damage size quantification. Additionally, the overall dimension of the sensor itself can be adjusted to fit the specific location or structural element, as well as the mutual separation of the sensing conductive strips of the CCPS. Due to the need to place such sensors in different locations on the monitored component or object, it is necessary to elaborate a rapid prototyping algorithm that will enable the design of CCPS of various shapes and sensing capabilities.

## 3. Parametric Models of CCPS

The CCPS sensor is an innovative solution; therefore, conducting a series of material and structural studies is necessary to diagnose its capabilities and limitations. A simulation model was developed and used to identify its mechanical and electromechanical properties to reduce the number of experimental tests required for this determination. In turn, two parametric models of the CCPS sensor were developed to expedite the process of preparing the computational model.

During the simulation studies, two computational software packages were used: ABAQUS and COMSOL Multiphysics. Both COMSOL and ABAQUS are among the most widely used finite element analysis (FEA) software in the engineering sector. They offer powerful tools for simulating a wide range of physical phenomena, but they differ in their overall approach, capabilities, and target user base. Each of these software packages has its own strengths and weaknesses, and the choice of which one to use often depends on the specific requirements of the project.

The COMSOL software is designed for multiphysics simulations, allowing users to simulate a variety of physical phenomena, and most of all, couple these different analyses (e.g., structural mechanic with electrical analysis) in a single model to create a comprehensive, multi-domain framework. COMSOL is a multi-disciplinary simulation software that specializes in modeling and solving partial differential equations (PDEs), providing a flexible environment for modeling complex interactions between multiple physics. This integrated approach allows for seamless and simplified setup and the analysis of different kinds of features with a unified workflow.

ABAQUS is a general-purpose FEA software that is widely used for simulating complex engineering systems. It has a robust solver, which is known for its ability to handle large and complex models. It is a finite element analysis tool, focusing on structural analysis, including linear and nonlinear problems. It excels in simulating complex material behaviors, contact problems, and dynamic analyses. ABAQUS uses an implicit solver for static and some dynamic simulations, and an explicit solver for dynamic simulations involving short-duration, high-energy events. It offers sophisticated material models, including plasticity, viscoelasticity, and damage mechanics, making it suitable for detailed structural analysis.

**Figure 2:**
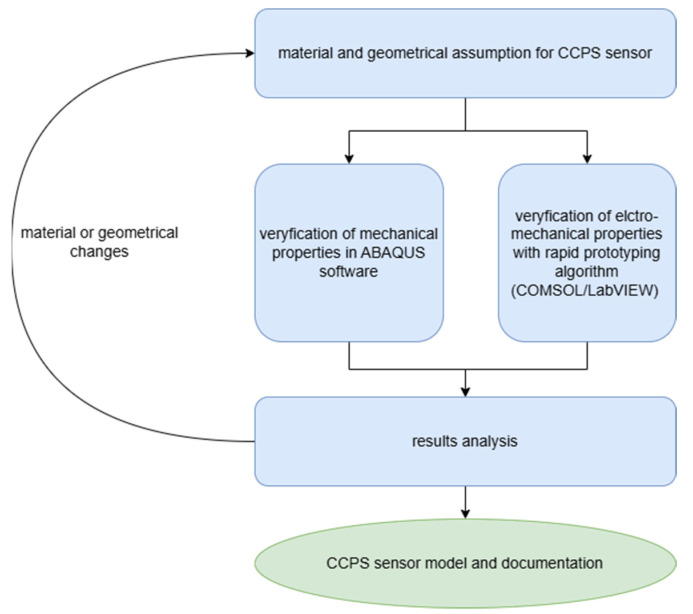
Flowchart of numerical model preparation for CCPS sensor analysis.

Utilizing both FEA environments, it was possible to perform comprehensive simulation analysis, leveraging the robustness of ABAQUS for calculation of a large model, as well as simultaneous multi-domain electromechanical research in COMSOL (see Figure 2).

The first CCSP sensor parametric model was developed with the use of a custom-made add-on called Detekta, based on the Abaqus software engine. This model was utilized to verify the sensor’s material and geometric parameters. The graphical user interface allowed for:

- Determining the impact of the sensor’s geometric parameters, such as the thickness of the contact layer and the top insulating layer, on the strain distribution within the sensor structure;
- Verifying the assumption that a thicker sensor structure may lead to delamination of the sensor from the tested surface;
- Checking whether there is a delay in the disruption of the sensor’s measurement grid relative to the tested structure as the thickness of the insulating contact layer increases.

The second parametric model was developed with the use of a rapid prototyping algorithm, and was developed in the COMSOL Multiphysics software. This algorithm enabled the configuration of geometric properties of the CCPS sensor and the preparation of its 3D model and technical documentation.

These two prepared parametric computational models of the CCPS sensor significantly reduced its prototyping time and the materials evaluation used for production of the CCPS. Additionally, they can be used by researchers who are less familiar with computational software. Both of the prepared models are described in detail below.

### 3.1. Parametric Model of CCPS Sensor Dedicated to ABAQUS Software

Due to the diversity and complexity of the real-world operating conditions of the sensors considered in the paper, a multi-stage approach was adopted for creating the simulation model. This approach involved using several simplified computational cases, each designed to analyze and verify one specific assumption. This method requires less computational power and shortens computation time compared to development of a comprehensive universal model.

Additionally, the parameterization and automatic generation of a series of calculations enable the rapid execution of multiple simulations to obtain a comprehensive set of results. This can lead to obtaining a broader understanding of sensor behavior under various conditions, which is crucial for the design and optimization process.

It is also worth noting that utilizing widely accepted methods for material property testing allows for easier comparison of computational analysis results with experimental data.

**Figure 3:**
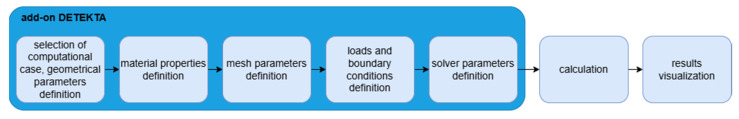
Flowchart of numerical model preparation in ABAQUS software.

To streamline and automate the modeling process in ABAQUS, the Detekta add-on was developed. This interface significantly accelerated and simplified the majority of steps involved in building the model, as illustrated in Figure 3. Moreover, it allowed for the creation of a database of material parameters, facilitating parameter management and enabling efficient reuse across different simulations.

**Figure 4:**
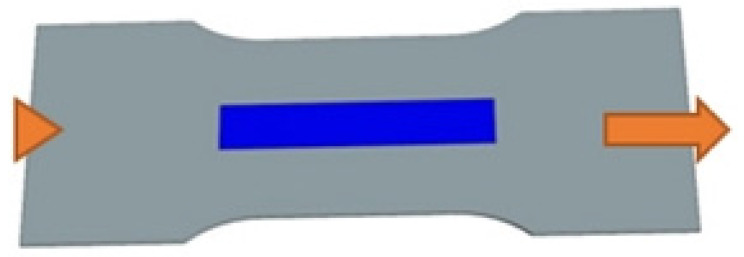
Diagram 1 of the computational case; tensile coupon test.

**Figure 5:**
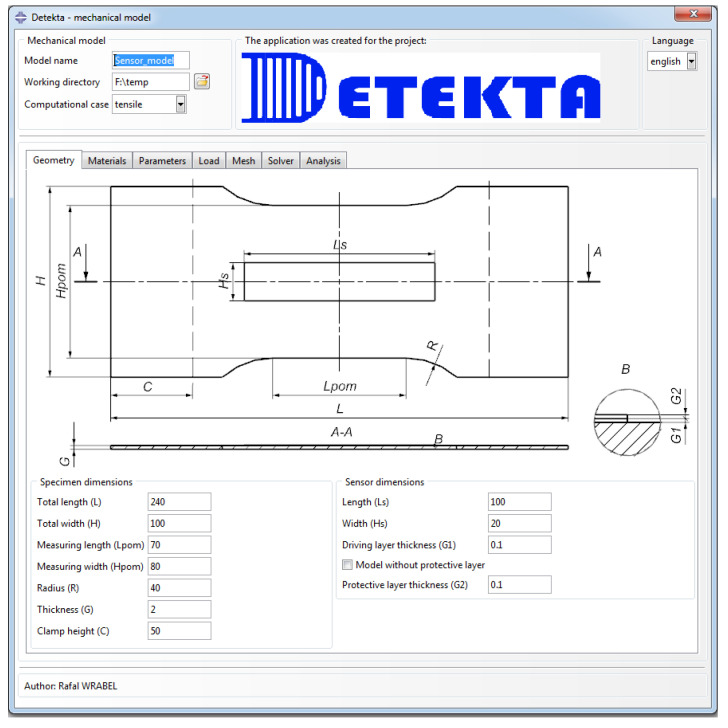
User interface dedicated to the definition of geometric parameters for tensile case.

**Figure 6:**
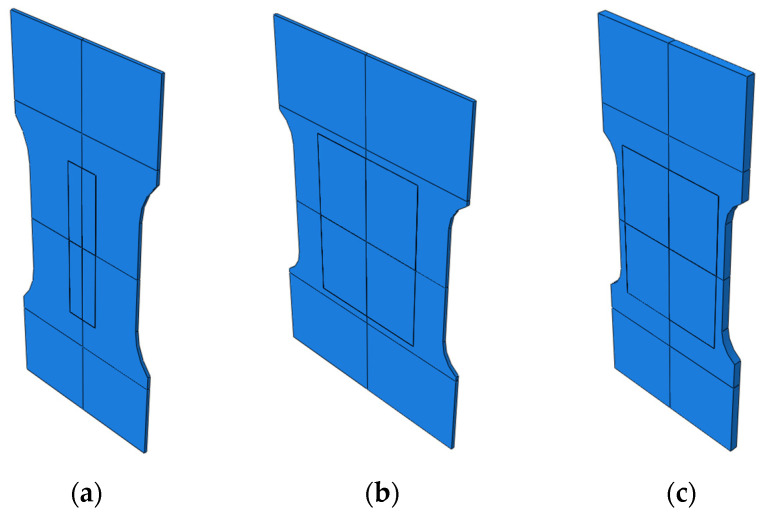
Different geometry variants for tensile test case: (**a**) variant 1; (**b**) variant 2; (**c**) variant 3.

The primary computational case in the mechanical model is the uniaxial tensile test of a flat coupon with an attached sensor (Figure 4). This case is mainly intended to study the impact of the sensor’s layer thickness on the strain distribution. As it was found that a flat rectangular plate sample would exhibit an uneven strain distribution under such loading conditions, a geometry with a constriction in the central part was designed. User interfaces dedicated to the definition of geometry parameters and its example variants are presented in Figure 5 and Figure 6, respectively. The key geometric dimensions for the default variant (Figure 6a) and the two additional shown cases (Figure 6b,c) are listed in Table 1.

**Figure 7:**
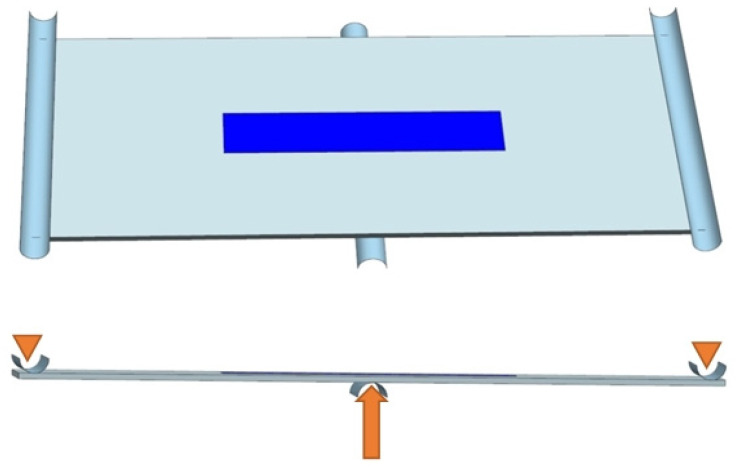
Diagram 2 of the computational case; bending.

**Figure 8:**
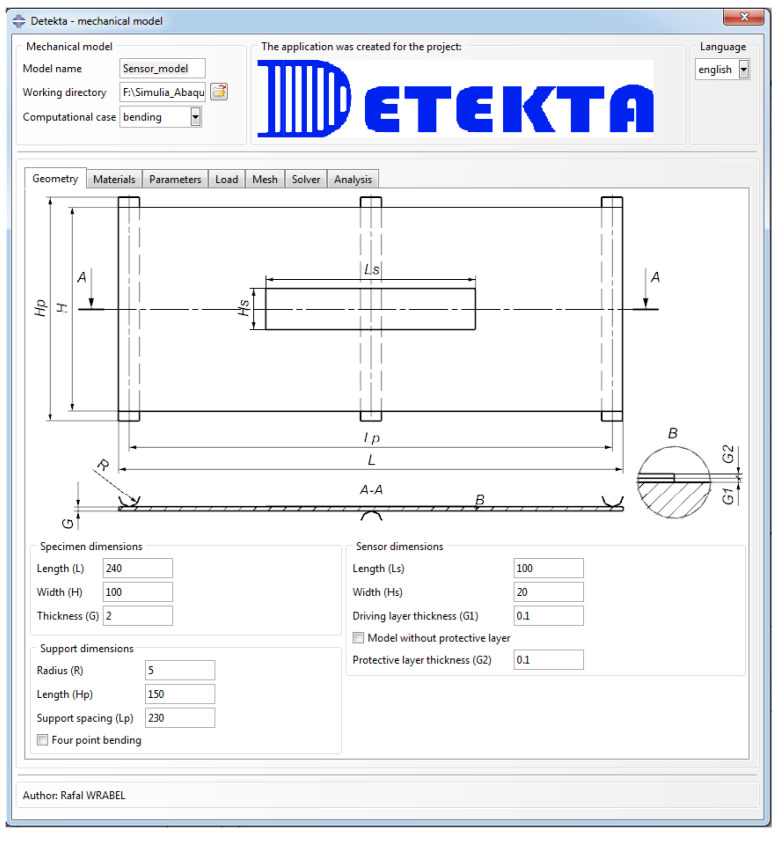
User interface dedicated to definition of geometric parameters for bending case.

**Figure 9:**
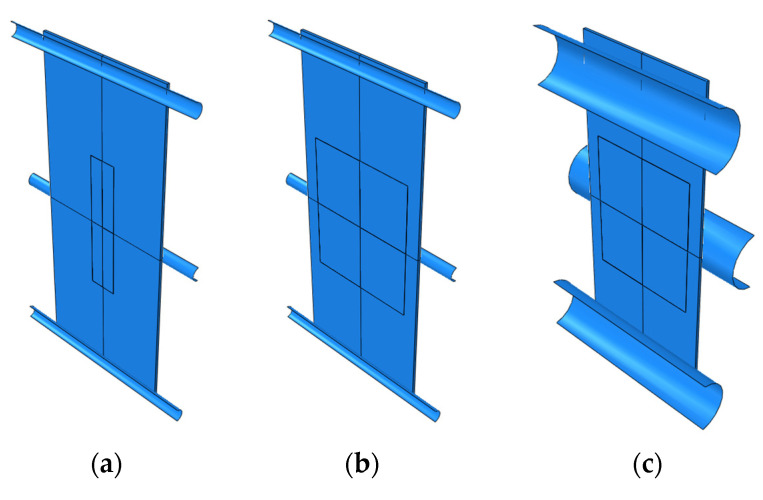
Different geometry variants for bending: (**a**) variant 1; (**b**) variant 2; (**c**) variant 3.

To test the sensor’s resistance to delamination from the host structure, a three-point bending case was designed using a flat sample with an attached sensor (Figure 7). This loading method is often used in studies to simulate detachment in a way where Mode I fracture (tensile cracking) predominates [22,23,24]. It is primarily applied in the study of composite structures, which are characterized by greater stiffness. However, the linear variation in the normal stress distribution within the cross-sections of bent elements provides the greatest likelihood of sensor delamination. The user interfaces dedicated to the definition of geometry parameters and its example variants are presented in Figure 8 and Figure 9, respectively. The key geometric dimensions for the default variant (Figure 9a) and the two additional shown cases (Figure 9b,c) are listed in Table 2.

### 3.2. Parametric Model of CCPS Sensor Dedicated to COMSOL Multiphysics Software

**Figure 10:**
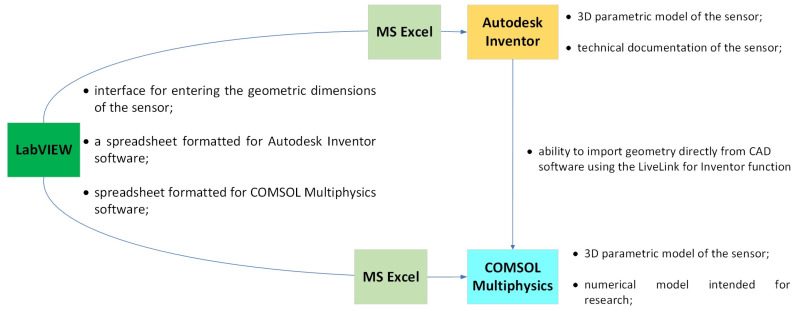
Rapid prototyping algorithm of crack detection sensor [6].

The previously described user interface was prepared as an add-on based on the Abaqus software to calculate the mechanical properties of the CCPS sensor. To conduct electromechanical studies, it was necessary to use software that integrates various physical phenomena. For this reason, COMSOL Multiphysics was employed, as it allows for mechanical, electrical, and thermal calculations, and others can be added if necessary. In this case, a dedicated user interface was also developed to further expedite the designing process. To prepare a model of a parametric CCPS sensor, a rapid prototyping algorithm was used, a diagram for which is shown in Figure 10.

The developed rapid prototyping algorithm of the CCPS sensor combines the functionalities of several engineering tools, including LabVIEW software with MS Excel, as well as Autodesk Inventor and COMSOL Multiphysics software. Using LabVIEW software, a user interface was developed for defining the geometric dimensions of the sensor. The prepared application also generated spreadsheets formatted in accordance with the requirements of Autodesk Inventor and COMSOL Multiphysics software. The first software mentioned was used to prepare a 3D model of the sensor and its technical documentation. Based on that, simulation tests for CCPS could be conducted in the COMSOL Multiphysics software.

**Figure 11:**
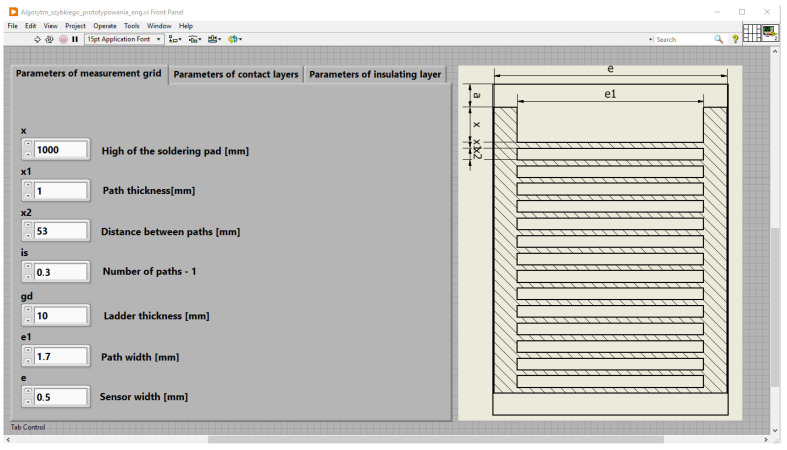
Application front panel designed in LabVIEW software [6].

The process of preparing a numerical model of the sensor using the rapid prototyping algorithm begins with entering its geometric dimensions using the user interface prepared in LabVIEW software (Figure 11). Based on data entered by the user, the prepared application automatically generates two spreadsheets formatted for Autodesk Inventor and COMSOL Multiphysics software. Files are prepared using the Express VI function called MS Office Report.

**Figure 12:**
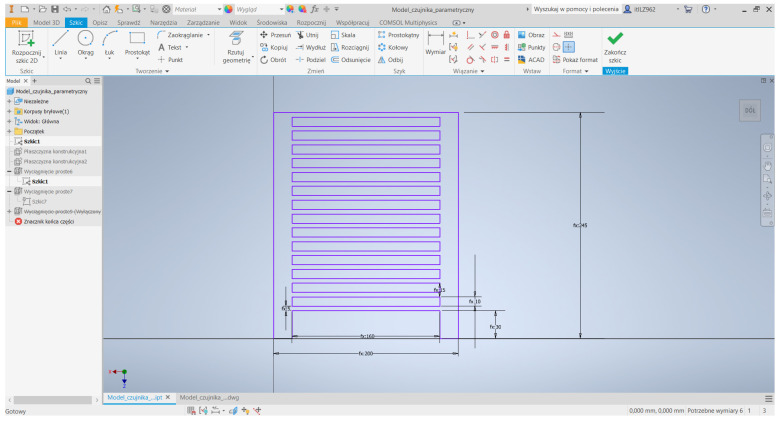
Model of CCPS made with use of Autodesk Inventor software [6].

The 3D model of the CCPS and its technical drawing prepared in Autodesk INVENTOR software are shown in Figure 12. The developed model can be used for numerical research in the COMSOL Multiphysics software.

Thanks to the LiveLink for Inventor library, it is possible to enter the CAD model of the test object directly into the numerical calculation program. In this way, the model preparation time is significantly shortened, especially when different variants of the shape and size of the measurement grid and sensor layers are considered.

## 4. Results of Numeric Calculation

The operation of the prepared parametric models was tested by performing mechanical calculations using the ABAQUS software, as well as calculations of electromechanical properties using the COMSOL Multiphysics software. With the use of the Abaqus-based software, the distribution of principal strains for bending and tensile tests were obtained. In turn with the use of COMSOL Multiphysics software, the total displacement field and von Mises stress were calculated, as well as electric potential distribution within the sensing layer (conductive grid) of the CCPS.

### 4.1. Results of Mechanical Calculation of CCPS Sensor with the Use of Abacus Software

Two example simulations were conducted to verify the functionality of the application dedicated to ABAQUS software, each using a different computational case and type of analysis. The assumptions made and the results obtained are presented below.

**Figure 13:**
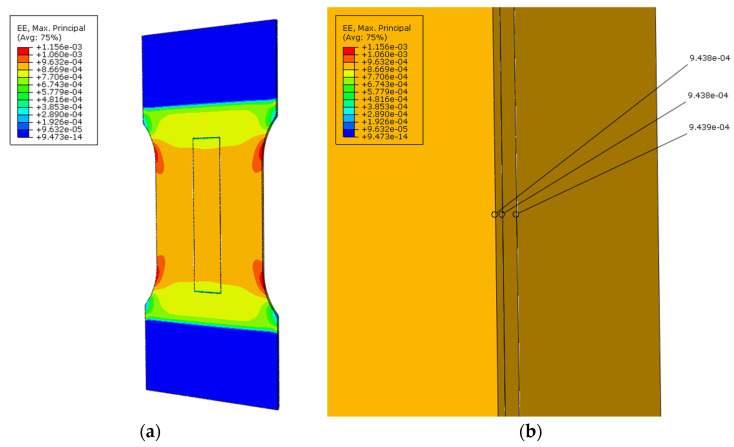
Distribution of maximum principal strains for the tensile case (insulation layer thickness 0.2 mm, axial force 10,000 N): (**a**) global strain distribution across the entire structure; (**b**) detailed view of strain concentration in the central region in the plane of symmetry.

**Figure 14:**
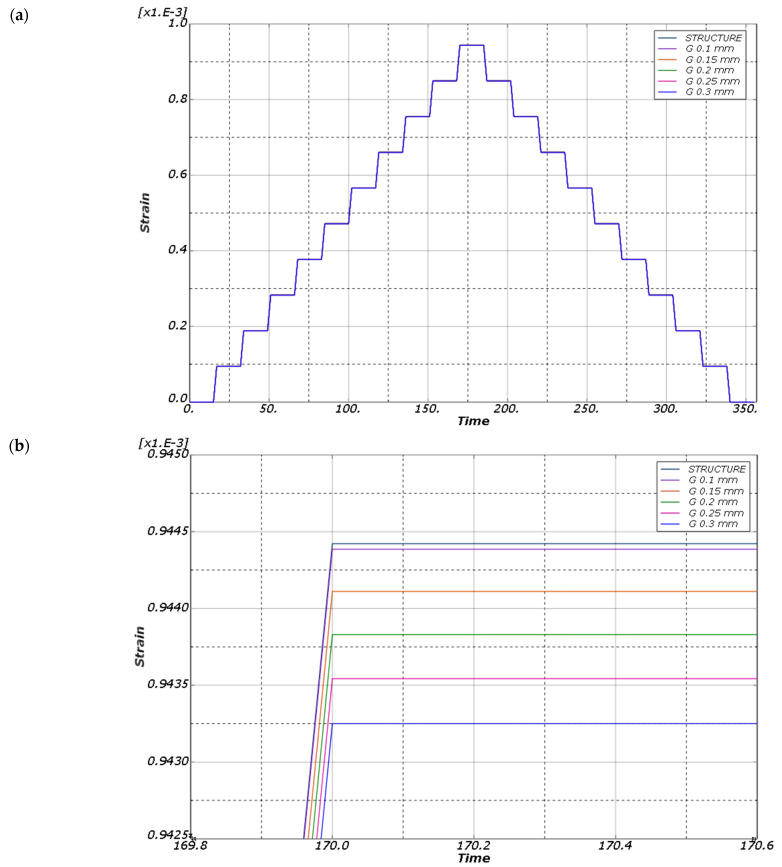
Graph of main deformations as a function of time for models with different thicknesses of the insulating layer: (**a**) the entire course; (**b**) values for the maximum excitation of 10,000 N.

The first computational case (the coupon under tensile) was used to test the option of generating a series of calculations. The thickness of the insulating layer, denoted as G, was varied in the range of 0.1 to 0.3 mm with a step of 0.05 mm, while the remaining geometric parameters were kept at their default values. Adhesive connections were not considered in the model as TIE [25] constraints between the sensor layers and the structure were used instead. This constraint type binds two separate surfaces together, preventing any relative motion between them, and allows the fusion of two regions, even if the meshes on their surfaces are dissimilar. The load was applied as a concentrated force, increasing incrementally from 0 to 10,000 N with a 1000 N step and then decreasing similarly back to 0. Each load level was maintained for 15 s. Both sensor layers were assigned a plastic material model of MG Chemicals 832C epoxy resin [26]. The calculations were performed using the “static” solver. The strain map for the maximum load value is shown in Figure 13, while the strain profile measured at the center of the upper surface of the insulating layer is shown in Figure 14.

**Figure 15:**
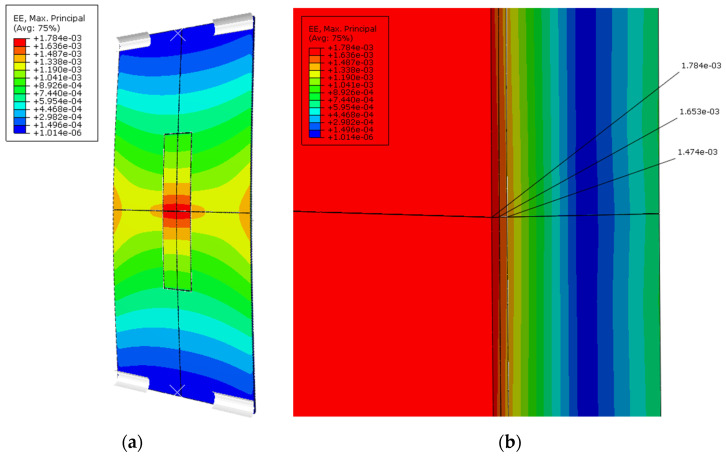
Distribution of maximum principal strains for the bending case: (**a**) global strain distribution across the entire structure; (**b**) detailed view of strain concentration in the central region in the plane of symmetry.

**Figure 16:**
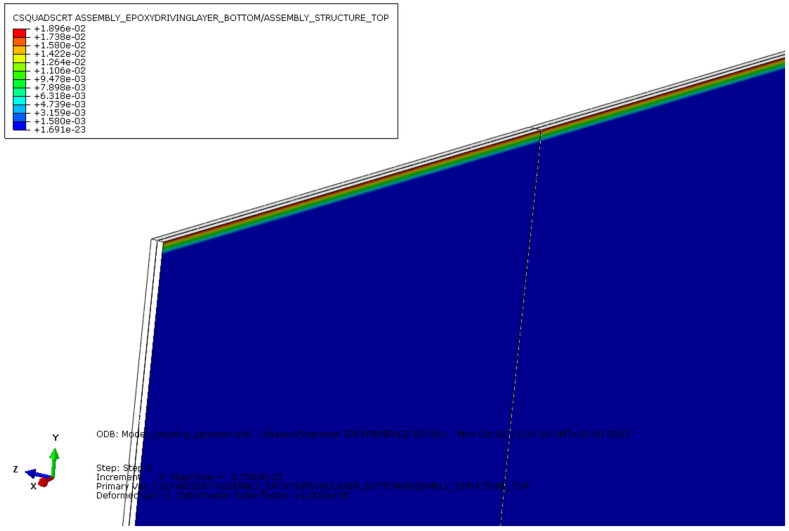
Visualization of failure criterion for the CZM connection (CSQUADSCRT).

The second example involved performing a single analysis for the three-point bending case. The same material models and solver as in the previous example were used. A new load amplitude was defined, with a linear increase from 0 to 2000 N. A CZM [27,28,29] was applied to represent the adhesive connection between the structure and the sensor. Figure 15 shows the resulting strain distribution. Figure 16 graphically illustrates the risk of sensor delamination from the structure.

### 4.2. Results of Electro-Mechanical Calculation of CCPS Sensor with the Use of COMSOL Multiphysics Software

As in the previous case, the sensor parametric model dedicated to COMSOL Multiphysics software was used to perform calculations of the mechanical and electromechanical properties of the CCPS sensor.

**Figure 17:**
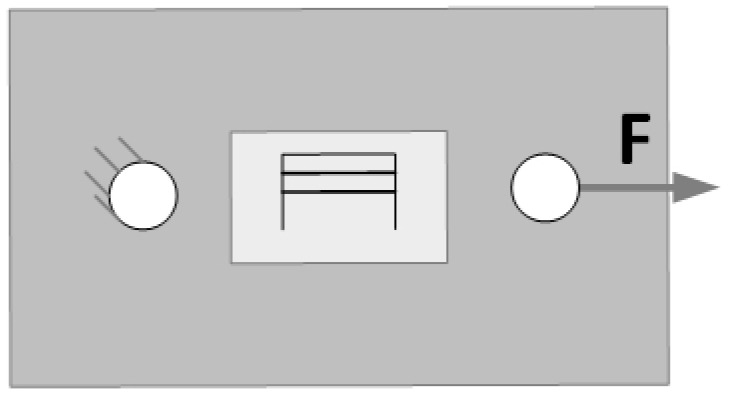
Schematic diagram of the numerical model used for mechanical studies with the use of COMSOL Multiphysics.

In the initial stage of testing, a study of phenomena related to the mechanical aspect of the sensor was carried out. In order to confirm the assumptions made and the correctness of the preparation of the numerical model of the sensor, a simulation of a static tensile test of a rectangular specimen made of aluminum alloy with the sensor glued on was carried out. A schematic drawing of the adopted numerical model and the conditions of the simulation are shown in Figure 17.

**Figure 18:**
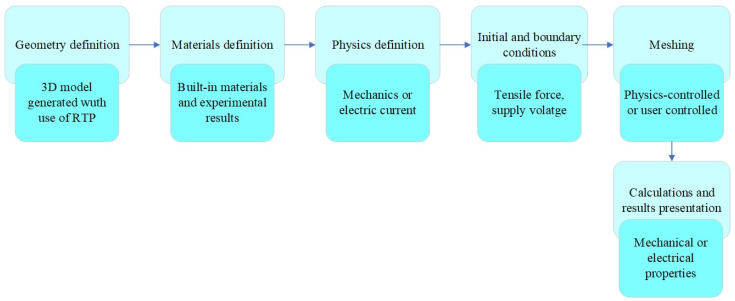
Flowchart of numerical model preparation in COMSOL Multiphysics software.

The sample shown in Figure 17 was rigidly fixed to the substrate on one side, while a load of 1 kN was applied on the opposite side, expressed as a total force. The model prepared in this way was used to perform static simulation tests, thanks to which the distribution of displacements and stresses occurring in the sample and the sensor mounted on it were obtained. The stages of preparing the numerical model for simulation tests are shown in Figure 18.

In the first stage of preparing the numerical model of the sensor for testing, its 3D model was prepared using LabVIEW and Autodesk Inventor software and imported to COMSOL Multiphysics. In the next step of preparing the numerical model for testing, its individual components were given material properties. The structure of the specimen was given the properties of aluminum alloy 2024 (UNS A92024), while the contact layer was given the properties of epoxy resin. The selected materials were imported from the material library of COMSOL Multiphysics software. They were not assigned the parameters necessary to perform calculations related to the mechanical phenomena taking place during the static tensile test (density, Young’s modulus, and Poisson’s ratio). To complete them, in the case of the 2024 alloy, tables of material properties available on manufacturers’ websites were used, while in the case of the resin, the results of the laboratory material tests were used.

Physics definitions were made after assigning material properties to the various elements of the model. Next, the initial and boundary conditions of the simulation were defined. It was assumed that the load applied to the edge of the specimen was expressed as a total force. Its value was 1 kN. On the other hand, rigid attachment ties (fixed constraints) were imposed on the opposite edge. A computational mesh was applied to the prepared geometry in the final stage of developing the numerical model. At this stage of the research, a “physics-controlled mesh” was used, with a density of “extra fine”. In this case, 216,015 domain elements, 58,474 edge elements, and 1484 edge elements were obtained.

**Figure 19:**
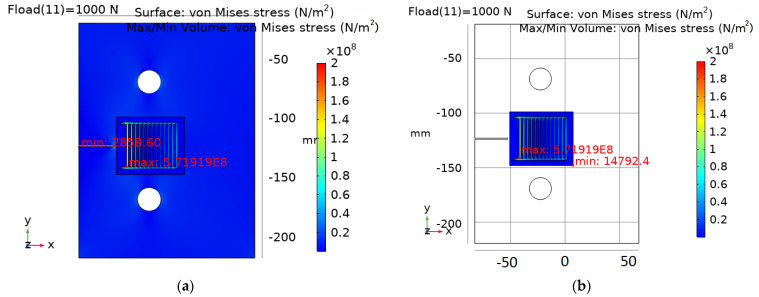
Calculation results of mechanical properties of the CCPS sensor, von Mises stress: (**a**) sensor with specimen; (**b**) layers of sensor.

The main goal of the analysis was to estimate the distribution of the stress field and displacements of the sample material and sensor layers due to pre-defined applied load. The solution was determined assuming constant forcing as a function of time. An example of sample calculation results is presented in Figure 19.

**Figure 20:**
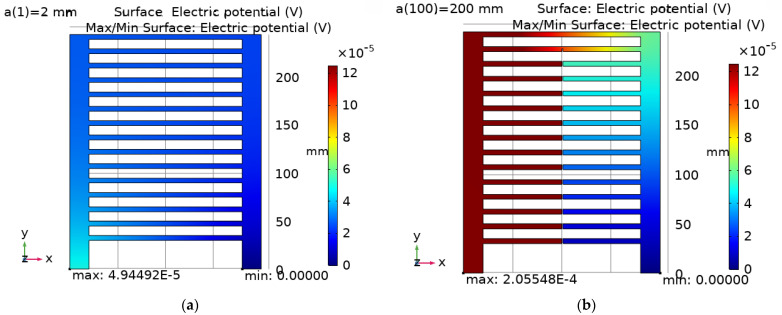
Distribution of the electric potential of the sensor’s electrically conductive layer: (**a**) 2 mm crack; (**b**) 200 mm crack.

In turn, the Electric Currents (EC) module of the COMSOL Multiphysics software from the AC/DC module group was used to prepare the electrical model of the CCPS sensor. In this case, the EC module was used to configure the electrical parameters and boundary conditions of the simulation. A stationary solver was used for these calculations. During the tests, calculations of the potential field of the sensors’ electroconductive grid were performed. Based on the simulation results, the electrical potential change as a function of the crack length characteristic was determined. Sample calculation results are shown in Figure 20.

During the simulation tests, the crack development was realized by cutting the sensor with a plane located in its axis of symmetry with a step of 2 mm from 0 to 218 mm. In Figure 20a, the size of the crack is 2 mm, so there is no crack in the electroconductive layer, and the maximum potential value is 4.95 × 10^−5^ V. On the other hand, in Figure 20b, the crack has reached a length of 200 mm, so the potential has taken the value of 2.06 × 10^−4^ V.

**Figure 21:**
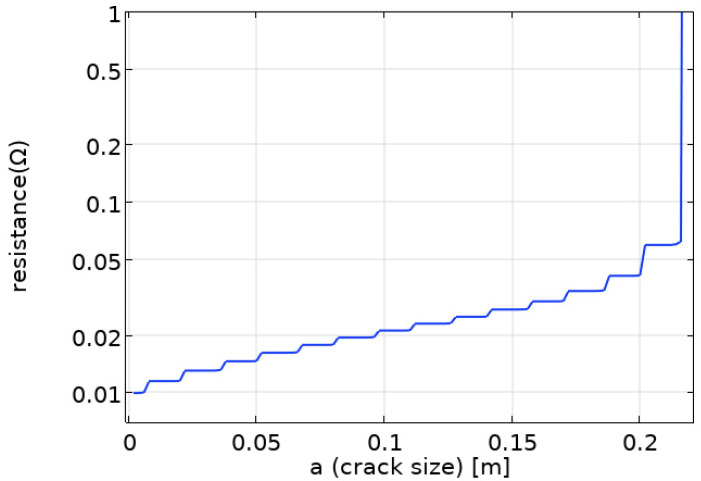
Change in the resistance value of the sensor’s electrically conductive layer with the development of the crack.

Additionally, the change in the resistance value of the CCPS’s electrically conductive layer caused by the cracking of individual sensing paths is shown in Figure 21.

## 5. Discussion

This paper presents computational verification of two parametric models of CCPS sensors. This new crack detection and propagation monitoring method presents enhanced functional features for SHM applications compared to commercially available crack gauges. The mechanic and electric properties of the new sensor type were obtained during simulation tests. The presented simulation test results were gathered via testing of the CCSP sensor with various geometric parameters, with the use of two separate computational environments.

The first part of the simulation studies was conducted with the use of a custom-made add-on based on the ABAQUS software engine, called Detekta. Two cases of mechanical impacts were considered: the static tensile case, and the three-point bending case.

In the first case, the global strain distribution (Figure 13a) demonstrated a uniform strain pattern across the central constricted region of the tensile sample. A detailed view of the strain distribution near the central axis (Figure 13b) shows minor differences in strain values across the layers: 9.438 × 10⁻^4^ at the top of the protective layer, 9.438 × 10⁻^4^ at the top of the driving layer, and 9.439 × 10⁻^4^ on the structure’s surface. In this case, the influence of the contact layer thickness on the stress distribution was analysed (Figure 14). The calculations suggest that, for the studied resin, the change in thickness did not significantly affect the strain distribution (differences are in the order of a few µStr) within the investigated range [30].

In the case of bending, the global strain distribution (Figure 15a) showed the highest strain values concentrated in the central region of the structure, corresponding to the region of maximum bending moment. A more detailed view of the strain concentration in the plane of symmetry (Figure 15b) reveals a sharp gradient, with peak values reaching 1784 [µstr] at the top of the protective layer, tapering to 1653 [µstr] at the top of the driving layer, and 1474 [µstr] on the structure’s surface. This rapid approx. 17% decrease in strain suggests a high risk of unwanted sensor surface cracking or delamination from the host structure of the contact layer. To analyze this risk, a Cohesive Surface QUADratic Stress damage initiation CRiTerion (CSQUADSCRT) [31] parameter was defined as the ratio of actual traction stress to the maximum value, meaning that delamination will occur when this parameter reaches a value of 1 (Figure 16). In this simulation case, delamination did not occur, but a sharp increase in this parameter can be observed at the sensor’s edge, which, with a thicker layer and higher structural load, could pose a risk of detachment. The software also allows for straightforward and time-saving analysis of the range of changes in a given material parameter, and an estimate of the impact of that change in the obtained results (parameter sweep analysis). All of this can be accomplished without advanced software skills, thanks to graphical user interface of the add-on.

In the second part of the study, a parametric model of the sensor, designed for use in COMSOL Multiphysics software, was utilized. During mechanical tests, distributions of von Mises stresses of the contact layer and electroconductive layer were obtained (Figure 19). The maximum von Mises stress value was found at the bottom edge of the electroconductive layer from the side of the rigid mounting of the specimen and was equal to 5.72 × 10^8^ N/m^2^. As expected minimum stress level was obtained at the pre-cracking part of the specimen. The simulation studies conducted using the first model confirmed the accuracy of the adopted numerical model. The calculations were performed using an automatic mesh in a very short time (under 2 min). In the case of a more complex geometry, especially involving the measurement grid, a user-defined mesh will likely be required, which would increase the computation time.

Additionally, using the COMSOL Multiphysics software, an analysis of the electrical properties of the sensor’s conductive layer was performed. Example distributions of the electric potential are shown in Figure 16. In turn, changes in the resistance value of the sensor’s electrically conductive layer with the development of the crack are shown in Figure 17. The changes in resistance occur discretely, which is associated with the breaking of successive sensor paths, and the sensor resistance characteristic is within the range of 10 to 60 mΩ for a designed layout. The shape of the CCPS characteristic is typical for a parallel combination of resistors, with a slight shift due to the position of connecting electrodes. After all the sensor paths are broken, there is a sharp increase in resistance, as shown in Figure 20.

## 6. Conclusions

This paper presents the results of theoretical research on the mechanic and electric properties of customized crack detection sensors. Due to the need to place CCPS in various parts of an airframe, it was necessary to develop a parametric model of the sensor along with a rapid prototyping algorithm that will enable the design of sensors with various geometric dimensions.

The prepared models of crack detection sensors can be used in their production process or during simulation tests to optimize the shape of their measurement mesh or the thickness of the sensor layers. Preparing a parametric model will significantly shorten the time of simulation tests, because in each iteration of testing, there will be no need to prepare another geometric model of the sensor, only to change the parameters.

The sensor models prepared using the rapid prototyping algorithm were utilized to conduct simulation tests in ABAQUS and COMSOL Multiphysics software. With the use of ABAQUS software, two computational cases modeled on uniaxial stretching and three-point bending were presented. These cases allow for determining the strain distribution within the sensor structure and its delamination from the surface. The additional add-on, based on the ABAQUS software engine, can be used to verify any user-defined material for the application of CCPS. The software also allows one to easily and quickly analyze the range of changes in a given material parameter, and estimate the impact on the change in the obtained results (parameter sweep analysis). All of this can be achieved without advanced software skills. In turn, with the use of COMSOL Multiphysics software, mechanical and electrical analyses of the sensor layers were conducted, and the distribution of von Mises stresses and electric potentials were obtained.

Prepared numerical models of the sensor were characterized by limitations resulting mainly from the limitations of numerical methods. The biggest problem here is finite computational resources, and the need to use a large number of degrees of freedom for more accurate calculations. Also important is the shape and size of the model’s mesh elements, over which more control can be exercised in Abaqus software. The COMSOL Multiphysics software, on the other hand, makes it possible to combine different physics of phenomena. For this reason, two different software packages were used during the simulation studies of the sensor.

Although the proposed crack monitoring method with use of CCPS sensors was experimentally verified, further research is still needed to increase its reliability and readiness for commercial use. In the future, experimental tests of crack detection sensors are planned, which will allow confirmation of the results of selected simulation tests and validate, e.g., the electromechanical model for its use in sensor output adaptation processes. Additionally, any uncertainties can be determined surrounding the estimated mechanical and electrical properties of the sensor.

## Figures and Tables

**Table 1:** Key geometry parameters for tensile case.

Parameter Name	Variant 1 (Default) [mm]	Variant 2 [mm]	Variant 3 [mm]
specimen total length	240	210	240
specimen grip width	100	130	100
length of measuring section	70	70	70
width of measuring section	80	110	80
radius of fillet	40	20	20
length of grip section (clamp height)	50	50	50
specimen thickness	2	2	5
sensor length	100	100	100
sensor width	20	70	70
driving layer thickness	0.1	0.1	0.1
protective layer thickness	0.1	0.1	0.1

**Table 2:** Key geometric parameters for bending case.

Parameter Name	Variant 1 (Default) [mm]	Variant 2 [mm]	Variant 3 [mm]
specimen length	240	240	240
specimen width	100	100	100
specimen thickness	2	2	2
support radius	5	5	20
support length	150	150	150
support spacing	230	230	230
sensor length	100	100	100
sensor width	20	80	80
driving layer thickness	0.1	0.1	0.1
protective layer thickness	0.1	0.1	0.1

## Data Availability

The data presented in this study are available on request from the corresponding author.
